# TeleHelp Ukraine: A distributed international telemedicine response to the ongoing war

**DOI:** 10.7189/jogh.14.04158

**Published:** 2024-10-25

**Authors:** Aditya Narayan, Mariia Petryk, Solomiia Savchuk, Katie Villarino, Ivan Lopez, Eva Morgun, Aleksandra Bakirova, Bohdan Kamets, Quan Le Tran, Sergey Komzyuk, Vrushali Kharbas, Steven Asch, Annalicia Pickering

**Affiliations:** 1 Stanford University School of Medicine, Palo Alto, California, USA; 2 George Mason University, Costello College of Business, Fairfax, Virginia, USA; 3 University of San Diego, San Diego, California, USA; 4 Northwestern University Feinberg School of Medicine, Chicago, Illinois, USA; 5 University of CA San Diego, San Diego, California, USA; 6 TeleHelp Ukraine, Stanford, California, USA; * Joint first authorship.

## Abstract

**Background:**

Humanitarian crises frequently garner solidarity and robust volunteer recruitment among health care communities. However, a common obstacle is matching providers to those in need across geographic and other barriers. We examined the application of a decentralised governance strategy in establishing an emergency telemedicine response, TeleHelp Ukraine (THU).

**Methods:**

Using a case study approach, we explored how global networking and technological advancements empower organisations to generate, access, disseminate, and utilise knowledge for sustainable health care delivery.

**Results:**

Preliminary results suggest that a non-profit, decentralised model strengthened by robust team dynamics may optimise the distribution of clinical workload and scheduling procedures. Institutional and cultural diversity among health care providers and volunteers fosters the mobilisation of knowledge resources, synergistic collaboration, and tailored care standards that align with both provider and patient expectations. By integrating these diverse, distributed networks, a synergistic effect is achieved, combining effective learning mechanisms with intellectual capital.

**Conclusions:**

Our study provides insights into the structure, implementation strategies, dissemination methodologies, and initial results of THU’s operation. These findings may inform future emergency telemedicine responses in humanitarian scenarios, thereby reinforcing the practical implementation of health as a human right.

The ongoing Russian war in Ukraine resulted in the displacement of vulnerable refugees and a catastrophic disruption in health care services within and beyond the nation’s borders, threatening the population’s right to care [1,2]. Within Ukraine, over 1000 health care facilities have been attacked since 24 February 2022, representing 15% of the country’s total health care facilities [3,4]. The consequently reduced health system capacity and damage to critical infrastructure worsen war-related illnesses, pre-existing medical conditions, and mental health [5–14]. As of 4 June 2023, there were more than 24 000 civilian casualties in total, including nearly 9000 deaths [15]. Providing critical services in this chaotic context poses ongoing challenges, made worse by the lack of international coordination of health services, unclear administrative guidance regarding health care access and refugee-specific protocols, and lack of training in requisite trauma-informed care practices [16,17].

In particular, fixed infrastructure in Ukraine, such as hospital facilities, water and power sources, and the like, are readily targeted by airstrikes and assaults [18]. To better match resource distribution to these areas of need, flexible approaches defined by distributed governance could help maximise the number and type of volunteers matched to specific local needs and overcome institution-specific roadblocks which may serve as a barrier to rapid, adaptable responses. Current initiatives to alleviate the crisis include public and private efforts to provide medical aid, material resources, and education dedicated to health system strengthening [19]. Additionally, several targeted efforts have been made to bridge gaps in services for high-needs patient populations, including those requiring complex neurosurgical procedures, as well as cancers and blood disorders [20,21].

Despite the robust and multifaceted international support for Ukraine, health care services remain insufficient. According to the Kiel Institute for the World Economy’s Ukraine Support Tracker, aid to Ukraine has included military, financial, and humanitarian assistance from 41 countries, including all EU member states, G7 members, and nations like Australia, South Korea, Turkey, and Norway [22]. The USA has provided approximately USD 75 billion in assistance, encompassing humanitarian, financial, and primarily military support. Further, international bodies such as the International Monetary Fund have offered significant loans to support Ukraine’s economic stability and provide a broad commitment to long-term recovery [23]. Finally, thousands of non-profit organisations, including the International Rescue Committee, the International Medical Corps, and Nova Ukraine, have mobilised to shore up gaps in social and health care needs. However, health-related humanitarian needs vastly range from the provision of first aid kits to emergency medical care in the near front-line regions, to ongoing unmet routine health care needs of those displaced but not in an active conflict zone.

As of April 2023, World Health Organization (WHO) surveys have highlighted persistent obstacles to adequate health care. For example, cost, time, and transportation difficulties were identified as key barriers to accessing medical care [24]. The availability of services and provider refusal significantly affected the provision of primary care and management of chronic conditions, while the high cost of medicines and treatment emerged as a major challenge for treating injuries. Medication accessibility improved between late 2022 and 2023, largely due to the humanitarian aid support, but the rising cost remained a challenge despite decreases in medicine shortages, pharmacy closures, and security concerns. Further, areas actively engaged in conflict and those outside government control reported lower access to primary care physicians and essential medications [25].

The surveys additionally underscore the heightened vulnerabilities faced by internally displaced persons, who exhibit restricted access to primary health care facilities and family physicians compared to the local population, alongside a heightened necessity to change family doctors due to displacement [24]. Security concerns and the absence of necessary documentation were particularly pronounced among this group, underscoring the complex challenges faced by this demographic in accessing health care amidst conflict. Finally, with respect to health status, chronic conditions were prevalent, with 42% of households reporting a member with such a condition [24]. Among these, cardiovascular issues were the most frequently cited, followed by diabetes and kidney disease.

To address barriers to care, the digital health landscape in Ukraine has been transformed significantly over the past years, especially due to the coronavirus disease 2019 (COVID-19) pandemic and subsequent military conflicts. Nevertheless, telemedicine adoption in Ukraine remains low. According to the recent United States Agency for International Development report, only 4.7% of visits are conducted using telecommunication methods such as teleconference, mobile phone calls, text messenger, and others [26]. The primary reasons for such low levels of use are the lack of technological infrastructure on both the provider and patient end, alongside a challenging regulatory and legislative framework that impedes wider adoption among providers. Thus, telemedicine projects in Ukraine have been limited and mostly originate from within the country. For example, one project established after Russia’s war against Ukraine facilitated psychological consultations by creating databases of doctors willing to consult online free of charge [26]. In another example, a large network of private clinics launched a chatbot in popular messenger for patient consultations [27]. Despite these efforts aimed at enabling greater access to health care since the Russian invasion, they represent fragments of the health care pipeline without providing the full continuum of health care. Therefore, limited options are available for patients who are less tech-savvy (e.g. for using chatbots) or who need more holistic examination by the health care provider (e.g. to overcome the limitations of text messaging).

Amidst the global humanitarian response to the war in Ukraine and recognising the urgent need to address gaps in health care services for war-impacted populations, TeleHelp Ukraine (THU) addresses these challenges by providing immediate medical and mental health support to affected populations. This organisation represents a unique, volunteer-led, international humanitarian response within a disaster context dedicated to increasing access to health care services to the internally and externally displaced Ukrainian population. Services provided by THU are distinguishable from other initiatives that serve Ukrainian citizens in implementing a low-cost volunteer operational structure, offering a wide range of health care offerings with a particular focus on mental health support and specialty care, and developing robust wraparound services through a health navigation programme. These combined efforts aim to bridge acute and persistent health care gaps, while ensuring that vulnerable populations receive necessary medical attention.

Here, we detail the structure, implementation, and dissemination methodologies that may facilitate future emergency responses via telemedicine in humanitarian contexts to operationalise health as a human right better.

## METHODS

### Operationalisation of care

**Figure 1:**
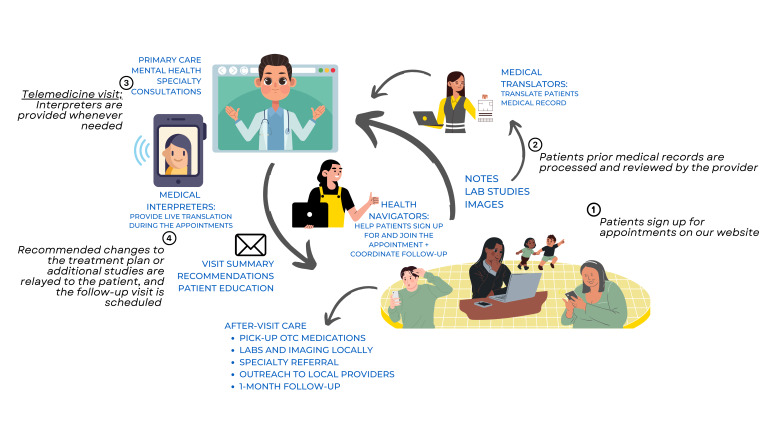
TeleHelp Ukraine core workflow. Patient registration: patients may sign up for a free on THU website. Appointment scheduling: patients choose their desired specialty, language, and time. Preparation: patients may submit prior medical records. Appointment for patients. Follow-up: written recommendations are shared with the patient.

THU offers a comprehensive range of clinical services through telemedicine for children and adults across various medical specialties such as psychiatry, cardiology, and oncology, among others. The organisation is powered by a global network of volunteer clinicians, case managers, and medical translators who facilitate health care delivery to patients. It currently has a network of over 200 volunteer providers and has facilitated over 1200 appointments within the first year following the full-scale Russian invasion of Ukraine. These volunteers are scaffolded by a secure telemedicine platform named ‘Cliniko’ [28], a dedicated patient-facing website [29], and a specialised communication network of volunteers. This integrated approach, which combines technical infrastructure with volunteer expertise, is foundational to THU’s operational model, promoting efficient patient care and wraparound care (**Figure 1**).

Four volunteer teams facilitate the telehealth operational model at THU. The ‘provider team’ consists of volunteer health care professionals (medicine, psychology, etc.) who offer medical consultations. The ‘interpreter team’ comprises medically literate volunteers with fluency in both the patient’s language (typically Ukrainian or Russian) and the provider’s language (typically English). They facilitate interpretation during live video appointments, translate patients’ medical records and documentation, and offer cultural brokerage by providing insights into the social and historical context surrounding patients’ concerns and conditions. The ‘provider services team’ consists of volunteers who assist providers in managing their availability and schedules, ensuring effective communication regarding workflow updates, provider outreach efforts, onboarding, and training. Lastly, the ‘patient services team’ includes volunteers (called ‘health navigators’) who facilitate communication between patients and health care providers regarding submitting and translating medical documentation pre-appointment, relaying provider recommendations, and scheduling follow-up consultations as necessary.

### Legal and privacy considerations

In response to the extensive destruction of health care facilities and the escalating demand for health care services, the Ministry of Health of Ukraine issued a special directive granting permission for foreign providers to offer telehealth services to the Ukrainian population within the territories of Ukraine.

THU’s *pro bono* legal counsel was secured via Hogan Lovells, an international law firm. Per their guidance, THU could operate with express permission from the government where the patient was located at the time of the visit, using the liability and standards of care defined by said location. Patient consent was also obtained at the time of the first registration. The THU team refrained from documenting patient location data in the telehealth records to safeguard patient safety amidst the ongoing and evolving precision shelling of Ukrainian cities by the Russian army. Emergency and immediate care protocols were also defined for concerns identified during telehealth visits that required urgent in-person response.

### Provider recruitment and onboarding

Provider recruitment was done through outreach to partner organisations, professional academic societies, social media, virtual events, and word-of-mouth. The provider’s identity and license were verified following the completion of an online application form. Identity verification was performed through the ‘Persona’ platform with the submission of a government-issued ID, while licensure verification was conducted using SheerID or a manual online search on government websites [30,31]. Provider eligibility was determined by active licensure to provide care in one’s country of practice. Once verification was complete, providers were registered on the ‘Cliniko’ platform where they could input availability, conduct virtual video appointments with patients, and write visit notes.

To enhance provider engagement and skill-building, THU organised virtual info sessions and webinars on trauma-informed care, conflict-context psychiatric care, and cultural sensitivity. These sessions provided a platform for Q&A and sharing experiences from existing THU volunteers. Engagement was further sustained through monthly updates, town halls for quality improvement discussions, expert-led clinical webinars, and feedback collection via anonymous surveys.

### Patient consent mechanisms and data privacy

The patient consent process was modelled after standard virtual clinic procedures, with additional emphasis on the international nature of the initiative and the use of medical interpreters. Prior to scheduling an appointment, patients were asked to provide consent electronically at the time of their first registration. The consent process focused on the general limitations of virtual visits, the use of medical interpreters, data privacy, and confidentiality parameters of the visit. As advised by the legal team, all clinical and non-clinical volunteers were required to furnish evidence of Health Insurance Portability and Accountability Act certification.

### Data analysis

We analysed the breakdown of patient care requested by the displaced population in Ukraine from May 2022 to May 2023. Primary data sources for our analysis included aggregate data from visits on the ‘Cliniko’ platform, traffic metrics from the landing THU website [29], online surveys of patient perceptions of care, and provider feedback via town halls. We omitted null responses from our analysis.

## RESULTS

### Recruiting volunteer providers

Over 438 providers submitted the THU provider application, of which 205 were onboarded over the first year of service. They were drawn from diverse geographic contexts, practice backgrounds, and language backgrounds (**Table 1**). Most provider volunteers were based in USA-based health systems (41.1%), private practices (21.6%), or mental health clinics or services (13.2%), while 10% were located in non-USA health systems.

**Table 1:** Provider characteristics (n = 205)

Provider characteristics	Number of providers
By institution	
*USA-based hospital*	84
*Private practice or self-employed*	57
*Mental health/therapy programme*	27
*Non-USA health system*	20
*Veterans Affairs*	3
*Not employed*	5
*Other*	9
By occupation/level of training	
*Mental health provider*	94
*Generalist*	82
*Specialist*	29
Speak and understand Ukrainian	
*Yes*	19
*No*	175
*Moderate*	8
Speak and understand Russian	
*Yes*	43
*No*	157
*Moderate*	5

VA –

Providers were mainly physicians (54.1%) and mental health service providers (45.8%). Among the medical providers, 73.9% were generalists (internal medicine, family medicine, emergency medicine) and 26.1% were specialists. A minority of providers are fluent or partially fluent in Ukrainian (9.3%) or Russian (21%).

### Reaching patients in need

**Figure 2:**
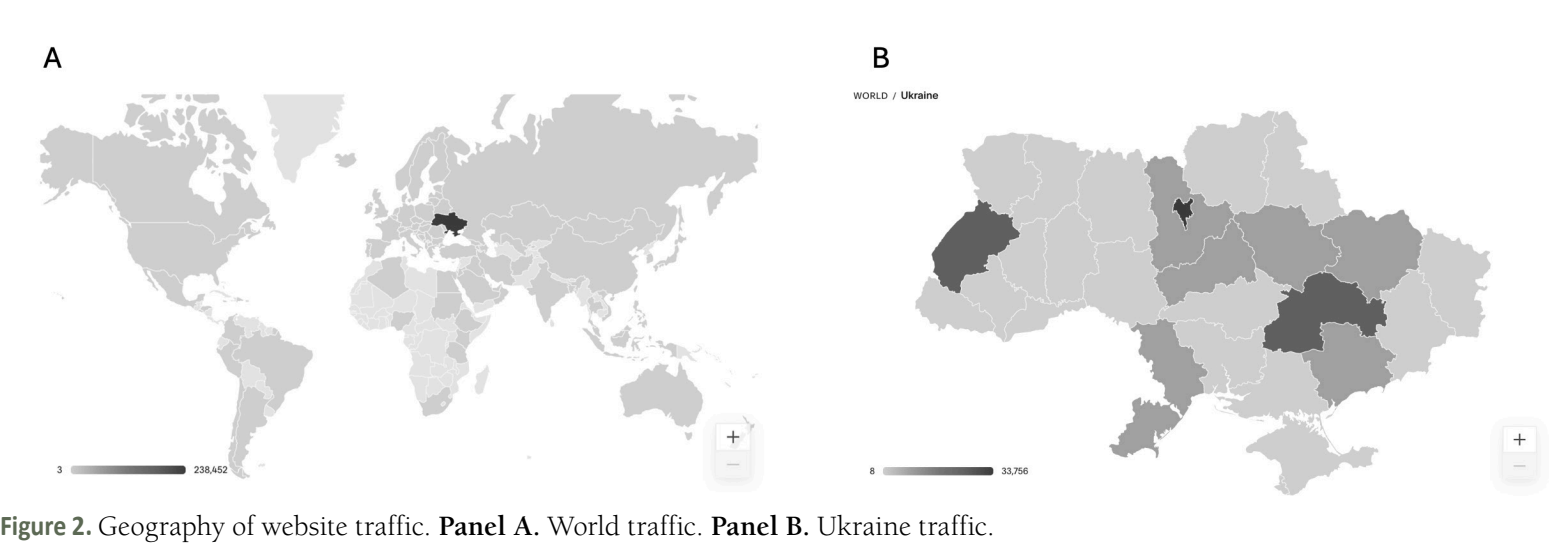
Geography of website traffic. **Panel A.** World traffic. **Panel B.** Ukraine traffic.

Over the first year of THU operations, the majority of those who visited the landing website were located in Ukraine (80.9%), followed by the USA (7.7%) and Poland (2.3%) (**Figure 2**). Among those in Ukraine, most were located in Dnipro, Kyiv, and Lviv, as well as other densely populated areas.

**Figure 3:**
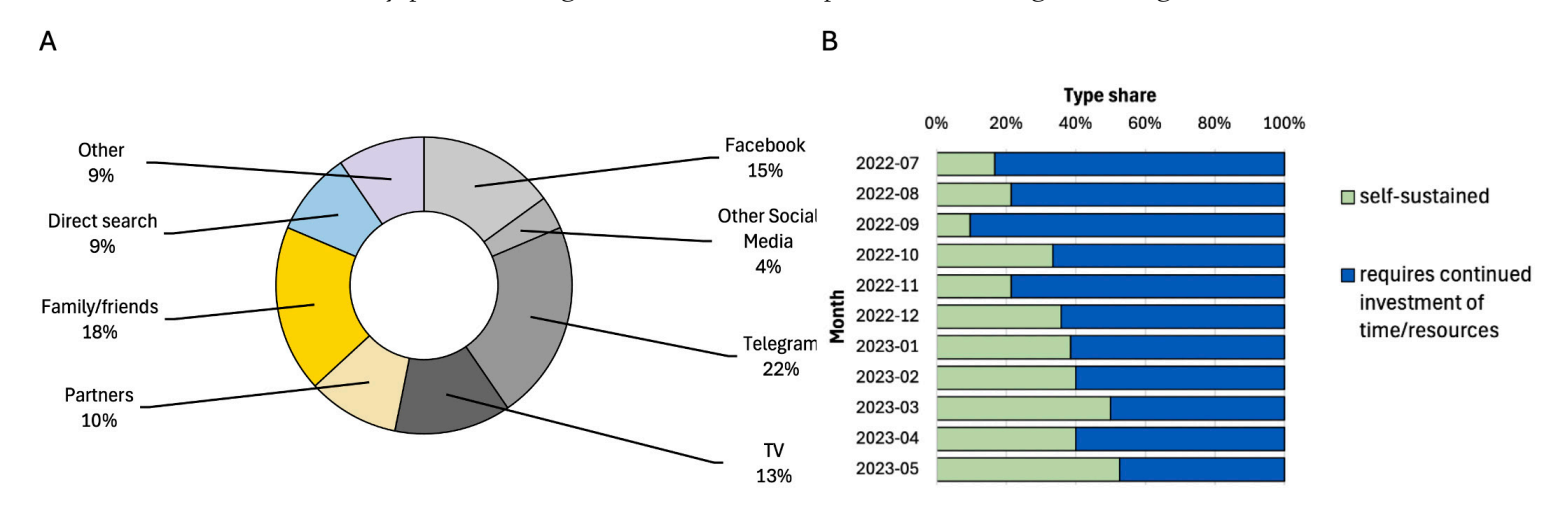
Effectiveness of patient outreach strategies. **Panel A**. By channel. **Panel B.** By the need of investment.

Based on the patient surveys completed following appointments, there was a range of methods through which they learned about the service. The most frequently reported avenues were Telegram – an encrypted centralised instant messaging service (21.8%), family/friends (18.2%), Facebook (15%), and television (12.8%) (**Figure 3**). Furthermore, an upward trend emerged over time, with a greater fraction of patients reporting the discovery of THU through avenues that do not necessitate continued investment (e.g. friends and family, direct search) (**Figure 3**, Panel A). In contrast, channels requiring investment (e.g. social media, messengers, television, partner organisations) saw a lower proportion of patient referrals (**Table 2**).

**Table 2:** Patient outreach strategies

Outreach site/venue	Mechanism	Impact on end-user engagement
Refugee shelters	Direct calls	Individuals often do not remain in shelters for long periods of time. It is difficult to track the impact of this intervention on service utilisation.
Facebook groups	Posting in groups for high-needs populations: refugees, parents, educators, etc	Varying impact on appointment scheduling by different groups. Many groups are challenging to access due to justified concerns regarding privacy and the necessity of relationship building.
Telegram groups	Posting in chat groups moderated by various entities (e.g. Ukrainian volunteer organisation, Nazar city bot, grunt) on the instant messaging platform, on Telegram	Consistent website traffic and patient flow. Posting in groups by the regions most impacted allowed for targeted outreach.
YouTube	Reaching out to existing YouTube channels with a significant following to share informational flyers and other information	Supported with generating public buy-in and visibility but did not lead to significant patient flow.
Traditional media outreach	Airing interviews on national and regional TV stations; national and local newspapers; radio; USA-based TV stations; and newspapers	Has led to significant spike-like increases in website traffic and appointment booking. May vary depending on the times of the day the story is featured on the channel or media outlet.
Partnerships with medical organisations	Partnering with individual physicians, clinics, non-profit organisations, and academic societies to share information on TeleHelp Ukraine services	There was minimal engagement from pharmacies and insurance providers. Greater engagement was identified with rural health clinics relative to large health centres.
Partnership with humanitarian organisations	Partnering with local and international non-profit organisations such as Nova Ukraine, Soborna Ukraine, Ukrainian American House to share information on TeleHelp Ukraine services	Supported with volunteer recruitment and additional partnership building.
Informational platforms and resource databases	Disseminate information about TeleHelp Ukraine through refugee resource databases and directories in Ukraine and Poland (palyanytsya.info, mapujpomoc.pl)	Challenging to gauge precise traffic generated from informational platforms.

### Utilisation of services

**Figure 4:**
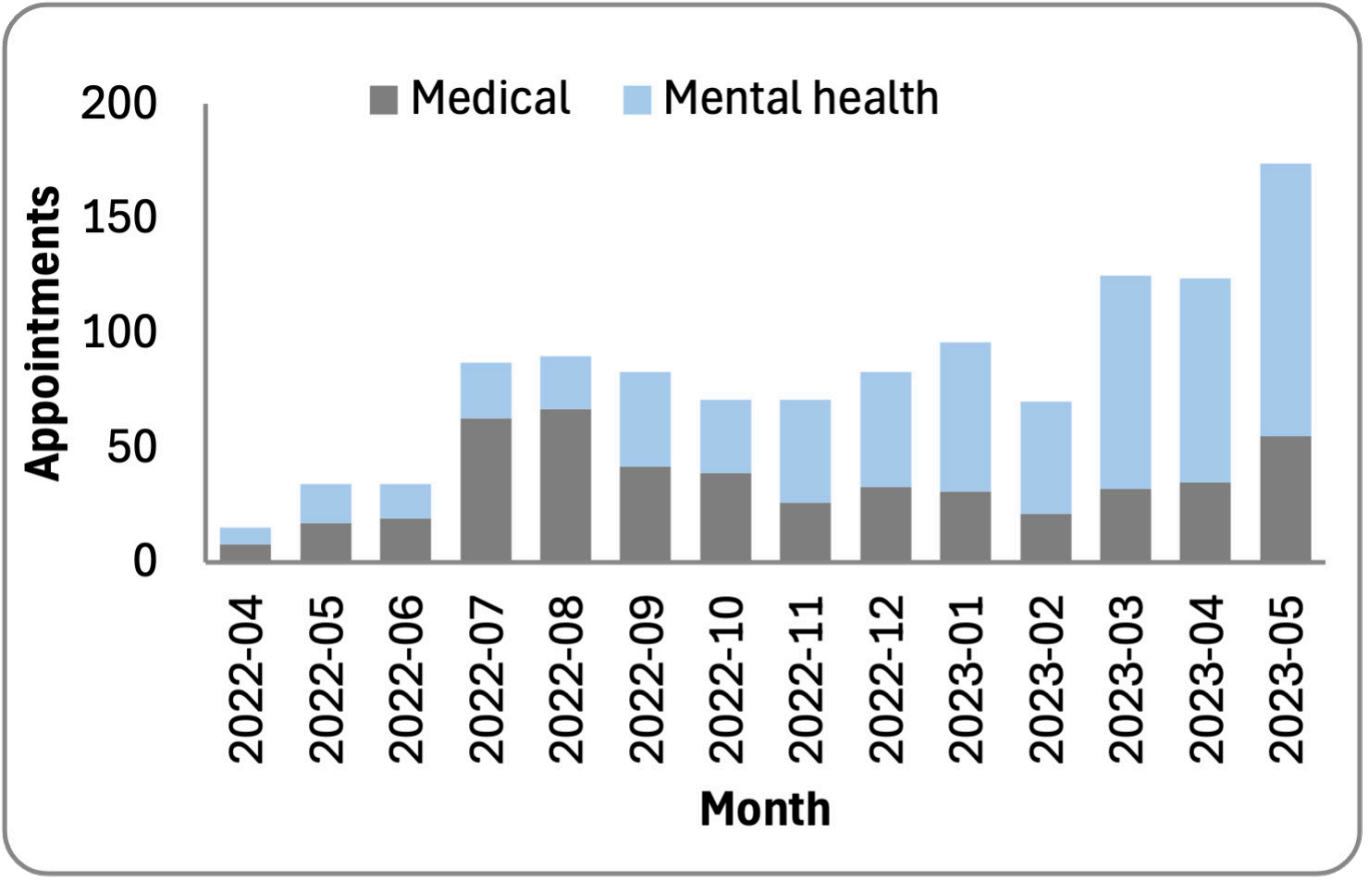
Completed appointments.

In its first year, THU conducted 1204 virtual appointments: 733 for mental health and 472 for medical consultations (**Figure 4**). There was a statistically significant trend observed in the increase in mental health sessions relative to medical appointments (*P* < 0.0001, χ^2^ trend test).

**Figure 5:**
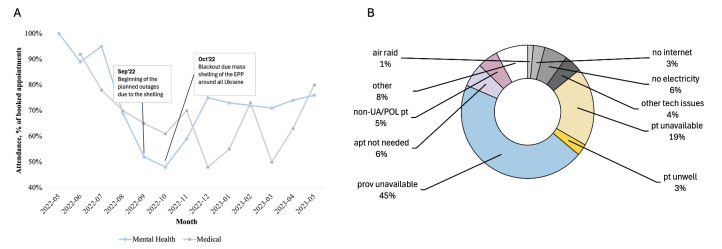
Appointment attendance. **Panel A.** Dynamic of attendance. **Panel B.** Reasons for unattendance.

To gauge THU service utilisation, we monitored scheduled, attended, and missed appointments (**Figure 5**, Panel A). From May 2022 to 2023, THU’s health care professionals volunteered over 2400 hours, of which 1600 were booked by patients, reflecting a 33% booking rate. Mental health services reported an 84% usage rate, contrasting with medical services at 19%. This discrepancy may be attributed to the surplus availability of medical specialists compared to mental health providers, with medical specialists averaging six hours of availability per month against mental health providers offering an average of two hours.

Our analysis of appointment attendance dynamics showed that the attendance rate remained high across the study period (**Figure 5**, Panel A). Appointment attendance decreased during the fall months, particularly in October 2022 (49%). Notably, beginning in October 2022, Russia began actively shelling Ukrainian energy infrastructure, causing power outages in many households [32]. To investigate the role that this factor played in appointment attendance, in October 2022, we began tracking the reason why patients cancelled or missed appointments (**Figure 5**; bottom). For appointments where reasons for non-attendance were available, those related to power outages (‘no electricity’, ‘no internet’, ‘air raid’) were the most cited reasons.

Among all appointments, neurology, endocrinology, and cardiology were the most utilised services (**Table 3**). Such evidence is aligned with the 2020 State of Telemedicine annual report from Doximity, which reflected similar high utilisation of these specialties among USA adults [33].

**Table 3:** Service utilisation by specialty

	Number of total appointment utilisation for specialities					
	**Cardiology**	**Dermatology**	**Endocrinology**	**Neurology**	**Rheumatology**	**Women’s health**
**Total appointments offered**	19	147	30	34	114	55
**Scheduled appointments (% of offered service)**	9 (47)	49 (33)	20 (67)	33 (97)	21 (18)	19 (35)
**Attended specialty appointments (% of offered service)**	9 (47)	31 (21)	14 (46.7)	29 (85.2)	15 (13.1)	17 (30.9)

### Patient perceptions of care

**Figure 6:**
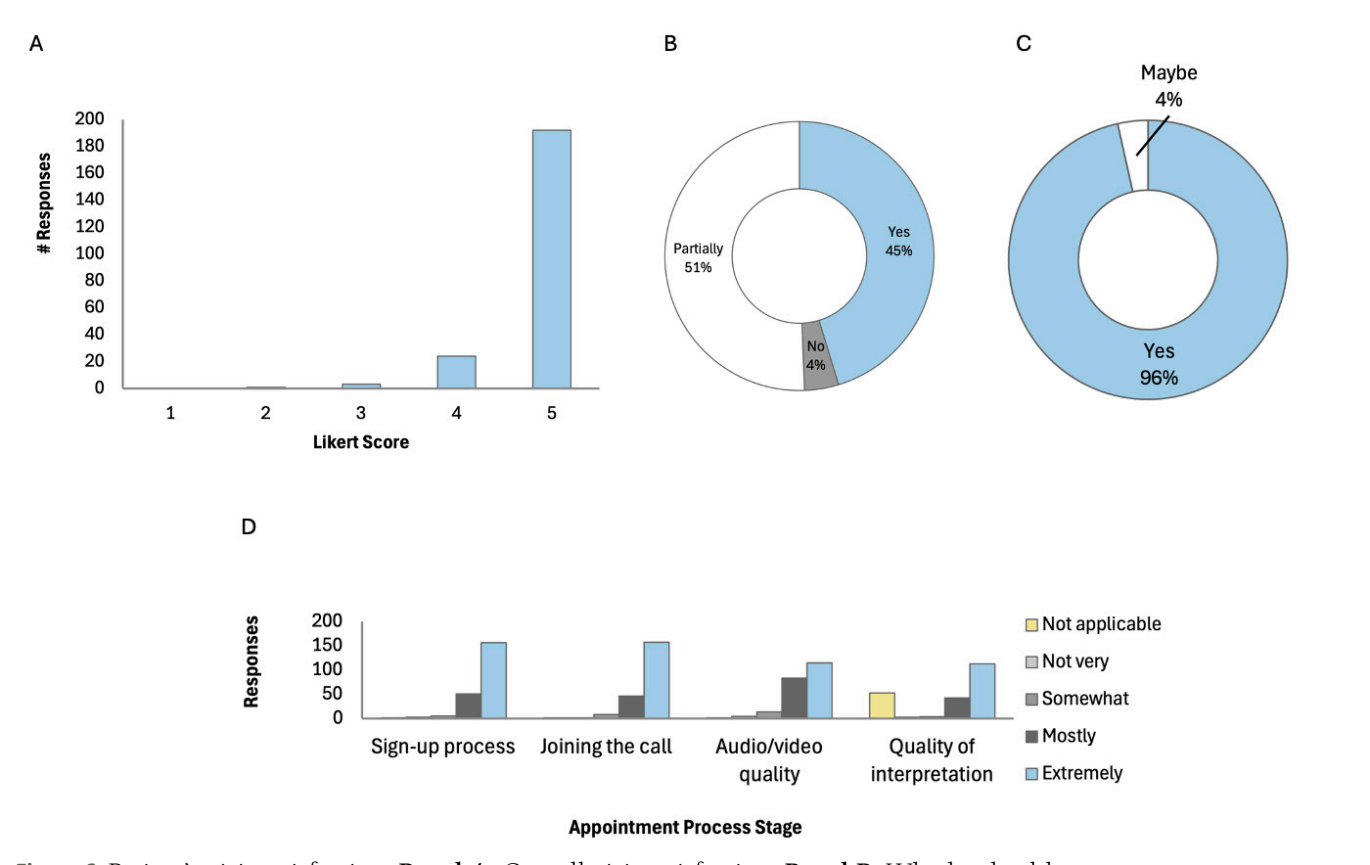
Patient’s visit satisfaction. **Panel A.** Overall visit satisfaction. **Panel B.** Whether health concerns were addressed during the visit. **Panel C.** Whether patient would recommend to friends/family. **Panel D.** Satisfaction level by the process.

We collected 220 surveys from patients who attended 944 appointments between July 2022 and May 2023 (23.3% response rate). The observed distribution of rating scores was J-shaped (**Figure 6**), as predicted by the literature, with an average visit satisfaction of 4.8 out of 5 points [34]. Most patients shared that their health problems were partially (55.8%), or completely (40.2%) resolved during their visit. Scores for satisfaction with the sign-up process (3.7/4), ability to join the call (3.7/4), audio/video quality (3.4/4), and quality of interpretation (3.7/4) were high, with 97.3% of patients being open to recommending the programme to others.

THU also administered a one-month post-visit survey. The response rate (9.4%, 57 responses from 606 appointments) was below the conventional boundary for survey representativeness (20%), therefore, significant underrepresentation biases might be present in the results. Among these responses, the majority indicate that they were able to follow their THU provider’s recommendations (68.5%) and that their health concerns improved (71.9%) or were resolved (18.8%).

### Operational challenges and solutions

To best support patients while maintaining provider engagement and flexibility, the THU patient services team began using Viber, a common texting platform in Ukraine, in order to send reminders to patients regarding upcoming appointments. The rate of ‘no-show’ appointments declined from 21% prior to the adoption of the tool to 14% after implementation. Although the rate of unattended appointments remained higher than the average reported in existing literature, particularly during the winter months (**Figure 5**, top), a majority of appointments were cancelled in advance rather than being missed altogether [35]. Furthermore, our investigation revealed that assigning a health navigator to each patient led to a decrease in the no-show rate from 26.2% to 18.2% (*P* = 0.011; χ^2^ test). Notably, patients had a higher likelihood of missing their initial appointment compared to subsequent follow-up appointments (30.8% vs 11.2%, respectively; *P* < 0.0001; χ^2^ test).

## DISCUSSION

In this study, we present a distinctive distributed model of health care delivery that connects an international network of volunteers and war-impacted populations in Ukraine amidst an ongoing humanitarian crisis. The inability to access medical assistance due to the extensive ruination of on-the-ground health care facilities within a short timeframe underscores the urgency for a rapid telemedicine intervention [36–39].

Several critical facilitating factors have contributed to the success of THU health care operations. First, the engagement of a globally distributed team of volunteers proved instrumental in operationalising services. Previous scholarly works explore the impact of distributed governance models on organisational engagement and effectiveness within the global health care landscape [40,41]. Additionally, THU’s institutional diversity granted access to a wide range of clinical professional networks. Second, THU offered a streamlined onboarding process and internal referral mechanisms. Notably, primary care-related providers demonstrated a higher likelihood of engaging in the THU initiative, with the collaborative referral pathways to specialist care further improving the quality of services provided. Third, we recognise the critical role of interpreters in international virtual health care contexts supported by a robust recruitment and training process. Patients demonstrated high satisfaction with interpreter services, which is in line with previous research establishing the potential cost-effectiveness and improved quality of care associated with telemedical interpretation [42–44]. Fourth, we showed that patients were broadly satisfied with their care, with most indicating that their health needs were met. The ability to establish continuous patient communication to address ongoing health care needs, troubleshoot logistical concerns, and provide robust follow-up referrals through an established network of services is central to the organisation’s function. Lastly, the situation in Ukraine may have been particularly favourable for recruiting a multinational virtual medical workforce. The prominence of news coverage motivated volunteer providers, while the prewar medical infrastructure was more highly developed than in many war-impacted nations.

Ultimately, several key strategies employed by THU are applicable to different geographic contexts or organisational structures. Organisations benefit in drawing from a broad volunteer base, including health care professionals and those with lived experience of the context being served. It must be noted, however, that THU’s volunteer network was built upon foundational infrastructure and an organisational framework designed for long-term sustainability. Partnerships with academic institutions enrich this model by introducing trained volunteers and integrating iterative research practices. For future efforts, it is also necessary to establish early external partnerships to ensure compliance with local regulations, optimising efficiency and legal adherence. Further, for patients, clinical services alone are insufficient. In our experience, the role of health navigators has been critical in facilitating care coordination. This is especially vital in dynamic conflict zones. We hope that each element of our discussion in which we describe facilitating factors may serve as a practical ‘guide’ for other future initiatives within or beyond this context.

### Challenges and limitations

Telemedicine inherently presents challenges that can contribute to increased rates of iatrogenic complications, which we proactively sought to address:

#### Barriers to delivering follow-up services

Insufficient follow-up may lead to medication mismanagement, feelings of abandonment by patients, or harm associated with interruptions in care. To address these needs, the health navigators team maintained regular communication with patients to ensure appropriate connection to community-based services, increased health literacy, and timely procurement of medications. However, no-show rates remained high and follow-up services were limited. Future efforts should focus on concurrent partnerships with direct service providers in the patient’s context.

Additionally, ensuring follow-up communication was difficult, given regulatory compliance hurdles. It required months of iteration to identify secure data storage services, select the appropriate technology tools, and establish communication channels with patients in Ukraine. These steps were crucial, however, for safeguarding patient privacy and ensuring secure handling of medical information, underscoring the need for expertise in both health care regulation and technology.

#### Quality of care

Due to variable backgrounds, differing cultural contexts, and levels of training among providers across borders, it was challenging to readily evaluate quality-of-care standards. In response, THU sourced clinical experts to share resources regarding best practices. The team also partnered with health services and implementation science research efforts that take place with academic partners to inform quality improvement. It must be noted, however, that change management has been an ongoing challenge. Specifically, we have had difficulties with ensuring providers adhere to predefined standards of notetaking in the electronic medical records, which in turn creates challenges with evaluating the level of clinical consultation provided, predicted cost-savings, and care quality. The absence of a comprehensive cost-effectiveness analysis of the initiative contributes to the potential inapplicability of some organisational elements to certain centralised or commercial health care contexts.

#### Addressing power asymmetries

It is crucial to consider how international health care delivery may reinforce long-standing global power asymmetries [45]. THU embodies a conscious ethos of supplementing existing services, intending to effectively transition patients to local community-based services. This goal was achieved by maintaining strong relationships with local government, institutions, health systems, providers, and patients, ensuring efforts were geared towards effective resource-sharing that supported the sustainable development of Ukrainian communities.

#### Volunteer-led

Given that THU is a volunteer-led and operated endeavour, challenges existed in standardising volunteer experience levels and enforcing expectations comparable to those of paid employment, particularly over extended periods of time. THU sought to address these challenges by implementing a hierarchical organisational structure, providing timesheets for volunteers, and assembling a robust quality improvement research team. Despite this, maintaining a longitudinal volunteer infrastructure poses significant challenges, including ensuring sustained engagement and managing the availability of volunteers over time. This requires continuous recruitment efforts, effective training programmes, and strategies to maintain volunteer motivation, while balancing the diverse needs and commitments of a largely unpaid workforce. It will be necessary to continue to evaluate the sustained investment of donors and volunteers.

### Limitations and future directions

Within the limitations of this study, it is essential to consider the contextual specificity of THU implementation and its potential applicability to other settings. While THU’s model has demonstrated feasibility and effectiveness in the Ukrainian context, which is characterised by high provider engagement and supportive infrastructure, extrapolating these results to different environments requires careful consideration.

For example, there are several limitations regarding the interpretation of data collected by THU. Our study captures a brief phase of THU’s lifecycle, potentially introducing operational biases, with inevitable contextual shifts as the organisation evolves. Such contextual factors include fluctuating geopolitics, infrastructure challenges, shifting health care needs, and population migration in Ukraine and Poland. These, in turn, disrupt care, affecting the quality, timeliness, and reach of telemedicine services, thereby limiting evaluations of efficacy and utilisation over time. Additionally, despite the informativeness of the available data, the data collection may be limited in some areas, such as the measurement of patient satisfaction. For example, though the patient satisfaction survey response rate was reasonably high at over 20%, we recognise that further analysis can suffer from the patient response bias and the positivity bias. Moreover, the socio-economic data of patient populations was limited due to privacy-preservation considerations related to the direct threats related to potential attacks. A more nuanced understanding of our user demographic will undoubtedly inform and improve our service offerings. Finally, as providers participating in this programme predominantly come from primary care fields and psychiatry, the results might be confounded by a potential sampling bias shaped by the leadership team’s available networks.

As THU continues to evolve, compelling future directions for its work lie in strategic partnerships and geographical expansion. Specifically, the organisation aims to collaborate with established medical teams and potentially integrate its services with mobile clinics to enhance health care access in remote and underserved communities. This approach will allow THU to tailor its approach to regions in need, leveraging its existing telemedicine platform while incorporating a new set of interpreters and health navigators who are culturally and linguistically attuned to the target communities. Additionally, there is considerable potential for detailed analysis of provider notes, enabling the organisation to examine patient outcomes based on specific clinical conditions. Finally, studies into the value of the care delivered and the financial sustainability of the THU model will offer invaluable insights for optimisation, supporting global health care delivery that is both equitable and sustainable.

## CONCLUSIONS

We found that a telehealth service delivered to patients in a disaster zone is a feasible strategy with high patient satisfaction and improved self-reported outcomes. The high demand for medical appointments and even greater demand for mental health appointments signify the importance of telemedicine access for vulnerable populations during periods of conflict. This volunteer-distributed telehealth model could be adopted by other global health initiatives to create more entrenched links to their target populations and thus create more sustainable, adaptable interventions.
